# Strategic Learning Principles Are Related to Academic Scores for Doctor of Physical Therapy Students

**DOI:** 10.1007/s40670-024-02215-5

**Published:** 2024-11-15

**Authors:** B. Reynolds, C. Minahan

**Affiliations:** South College School of Physical Therapy, 400 Goody’s Lane, Suite 101, Knoxville, TN USA

**Keywords:** Study strategies, Physical therapist education, Hybrid learning, Self-regulation, LASSI, Strategic learning

## Abstract

**Introduction:**

The Learning and Study Strategies Inventory (LASSI) is a self-assessment of strategic learning principles with scores representing areas for growth. The purpose of this study was to measure LASSI scores at 2 points in time for Doctor of Physical Therapy (DPT) students in a 2-year hybrid program to see if scores changed after a science of learning course and two quarters of the program. The authors then examined the relationship between LASSI scores and academic scores (DPT program GPA, anatomy, and physiology grades).

**Methods:**

Retrospective descriptive analysis of LASSI scores with comparisons between baseline and after a science of learning course and two quarters of the program using a paired *t*-test; bivariate correlations examined the relationship of LASSI scores to academic scores.

**Results:**

Data was collected for 259 matriculated DPT students. Three of the 10 LASSI scales had significant change after the science of learning course and two quarters of the program; however, the effect sizes were small (0.2 or less). Anxiety management (*t*(258) = 2.340, *p* = .020) scores improved, while both concentrations (*t*(258) =  − 3.229, *p* = .001) and the use of academic resources (*t*(258) =  − 1.999, *p* = .047) had lower scores. There were several LASSI scores with significant correlations to academic scores (*ρ* = .132 to .431).

**Discussion/Conclusion:**

LASSI was related to academic scores, although the strength of the relationship was low to negligible. LASSI showed small changes over time. There could be various reasons for scores improving or declining, but the awareness of scores and change in scores can provide a solid foundation for individualized coaching to DPT students as they navigate the rigor of a graduate level professional program.

**Supplementary Information:**

The online version contains supplementary material available at 10.1007/s40670-024-02215-5.

## Introduction

Strategic learning incorporates individual motivation for learning and a learner’s intentional systematic approach to develop and transfer knowledge and skills as needed to reach learning goals [1]. Strategic learning is a component of the broader theory of self-regulation of learning: strategic learning principles help students self-regulate their learning. Self-regulated learning is considered a vital factor in achievement of academic outcomes [2–5]. Successful implementation of self-regulated learning strategies correlates with positive academic outcomes [4]. Learners who apply self-regulated learning principles take responsibility for the learning processes via metacognitive, motivational, and behavioral processes [6].

Self-regulation of learning, including strategic learning, can contribute to successful knowledge retention [6, 9]. Learners may underestimate the value of science-based learning strategies that can increase academic success; they may rely on learning strategies that could be less effective [10–13]. Self-assessment and coaching these learning principles may support student success. There are several reasons self-regulated learning is important for physical therapy students and other health education programs. Given the rigor of the first year of a Doctor of Physical Therapy (DPT) program (and likely other health profession programs), educators see students with academic challenges early in the program. Promotion of strategic learning and self-regulated learning principles through coaching/mentoring, learning plans, goal setting, or other interventions could positively impact the student experience and academic success. Pertinent to all healthcare education, fostering self-regulated learning can help support individual accountability to the life-long learning process and build the versatile and adaptive expertise needed to address the ever-changing demands of healthcare practice [7, 8].

Self-regulated learning and strategic learning are measured in different ways, often using self-report questionnaires. The Learning and Study Strategies Inventory (LASSI) is a self-report questionnaire that is used to broadly assess elements of strategic learning via ten scales, guiding learners in a reflection of strengths and weaknesses [14]. LASSI asks students to reflect on their thoughts, behaviors, and attitudes related to things like knowledge identification, testing knowledge, motivation for learning, and anxiety. LASSI scores may change over time and may be related to academic scores for professional healthcare students. In medical students, 5 of the 10 LASSI scales were significantly associated with anatomical science academic achievement and performance on the medical licensing exam [15]. Pucillo and Perez [16] used the LASSI and a metacognition self-report score to describe the predictive relationships between aspects of self-regulated learning and academic achievement in 75 DPT and occupational therapy students in a hybrid blending learning model. The LASSI scale of *Time Management* was able to explain 35% of the variation in first term academic grade point average (GPA). Students with scale scores below the 75th percentile for *Time Management* and *Concentration* were 5 × more likely to experience a GPA below 3.0 at the end of the first term, while those scoring below the 75th percentile for *Anxiety* management were 7 × more likely to have a GPA below 3.0 [16]. Other authors have examined LASSI scores in DPT students and looked at changes in LASSI scores at various points in time. Results indicated some scale scores improved (higher score) and some did not [17–20]. Several authors have examined LASSI scores in health profession students. Many included samples of medical students. There are several authors listed here that examined DPT students. Additional research is necessary to examine larger samples of students from a variety of programs and locations to support validity and transferability of findings.

Intentional practices and/or coaching may improve learning strategies and the use of strategic learning principles in professional healthcare students. One way to establish a baseline for use of learning strategies is through the LASSI. The purpose of this study was to first measure LASSI scores at 2 points in time in DPT students from a 2-year hybrid program to determine if scores changed after a science of learning course and two quarters of the professional program. The authors then examined the relationship between LASSI scores and academic scores (DPT program GPA and anatomy and physiology grades).

## Methods

### Ethics Statement

Expedited IRB approval was completed through the institution, #22–015.

### Study Design

This study is a retrospective descriptive analysis examining scores used within the program as part of the regular collection of data.

### Setting

Data was available to faculty in the program as part of regularly collected program data. The study was conducted at a 2-year hybrid DPT program in a private primarily nonresidential college in the USA.

### Participants

Data was analyzed for three cohorts of entry-level DPT students (*n* = 259) admitted to the program in 2020 and 2021 (two cohorts admitted in 2021).

### Program Course Structure

**Fig. 1:**
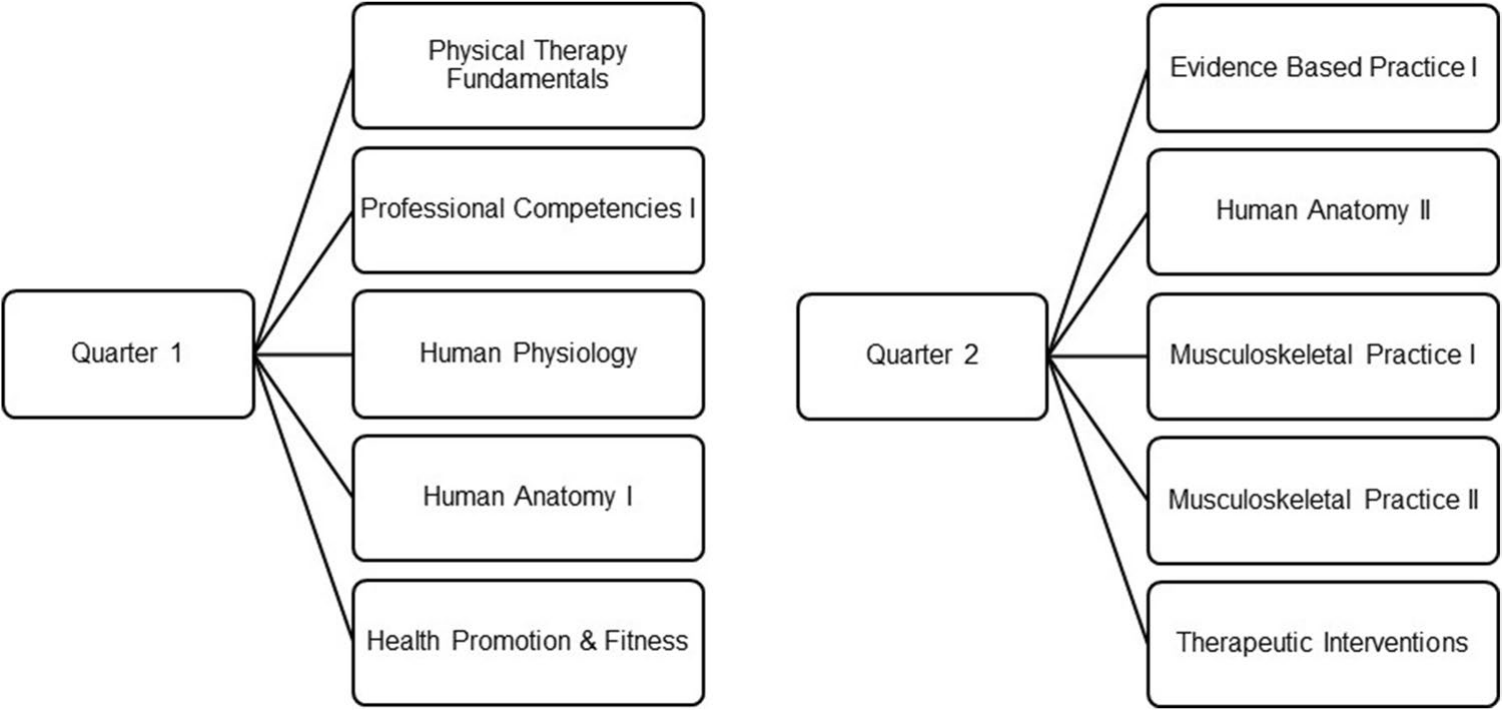
DPT program courses in Quarter 1 and Quarter 2 of an 8-quarter program

The first and second quarters of the 8-quarter program include foundational science courses, applied science courses, evidence-based practice, therapeutic interventions, and the first two patient management courses in the musculoskeletal system. Figure 1 outlines the courses in each respective quarter.

### Science of Learning Course

Students complete a baseline LASSI during orientation. This data is used in individual academic coaching meetings with faculty to discuss areas of growth. During orientation, students also complete a science of learning course after taking the LASSI. The asynchronous course is self-paced, expected to be completed over a period of 4 weeks before the first quarter commences, and has a focus on evidence-based learning principles. An initial module explains the neuroscience of how the brain learns and retains knowledge for long-term use and provides myth busters of commonly misunderstood perceptions of learning. The next module provides descriptions and specific illustrations of how to use concrete examples, dual coding, elaboration, interleaving, spaced practice, and retrieval practice while learning. A module on self-awareness with learning provides perspectives to enhance learning, including exercise, focusing techniques, sleep and stress management, an environment conducive for studying, and the impact of emotions on learning. The last module provides an introduction to the Master Adaptive Learner framework [21] with a description and examples of the self-regulation of learning via metacognition, curiosity, motivation, and a growth mindset to support adaptive expertise, clinical reasoning, and the process of life-long learning. There is no content in the science of learning course that specifically addresses the LASSI scale components or LASSI results. There is no assessment within the science of learning course.

### Data Sources

All data were examined retrospectively from the DPT program database and included data readily available and tracked as part of DPT program outcomes. While the data was initially recorded in an identifiable manner for program tracking and outcomes, researchers pulled data and removed identifying information for this research. The subjects were not contacted or re-identified.

**Fig. 2:**
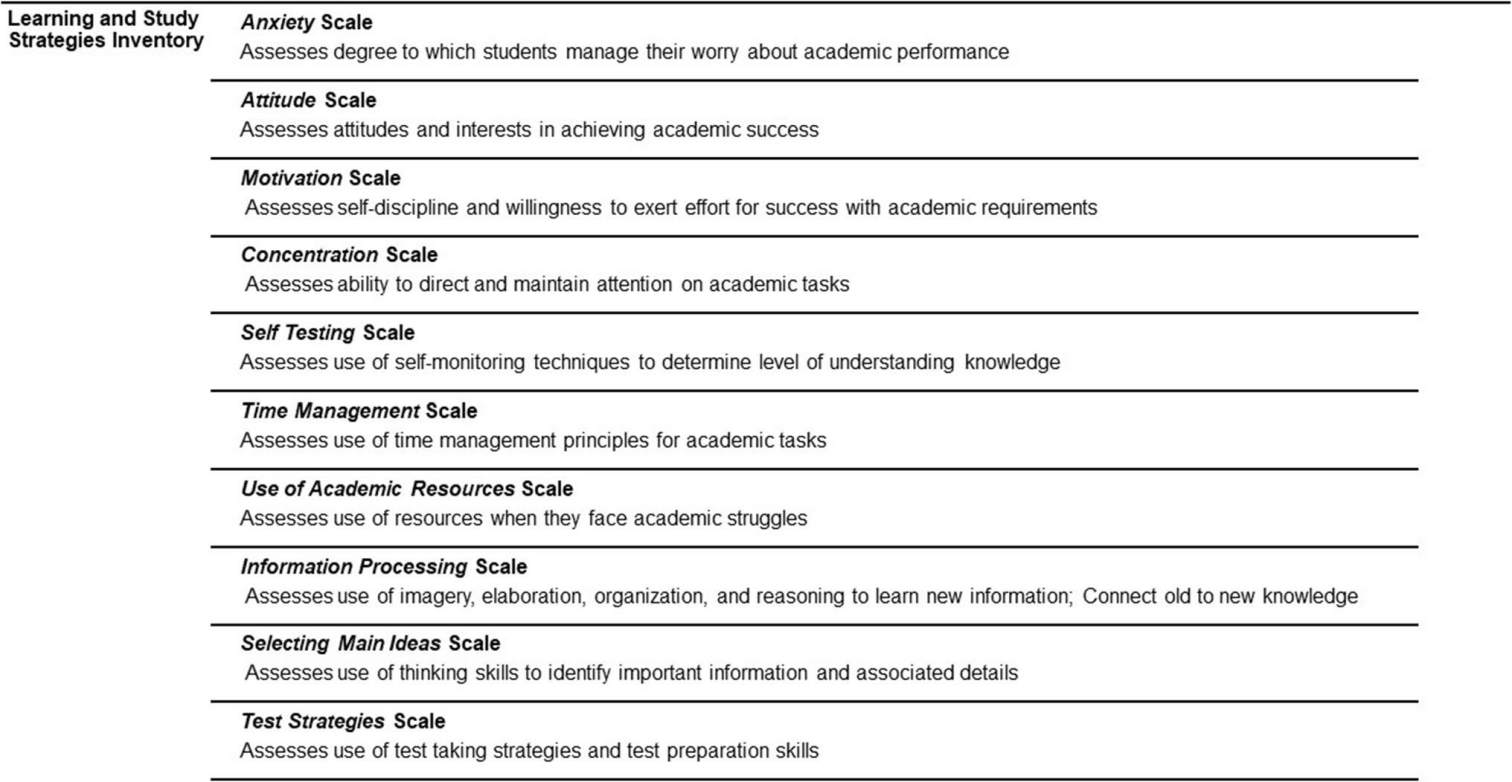
Descriptions of the 10 LASSI scales

The LASSI for Online Learning (LLO) is an adapted version of LASSI; this tool was used to measure strategic learning strategies. The online learning version [22] of this tool uses the same language but substitutes classroom environment language with home/remote learning. The same ten scales are used to summarize perception of specific learning strategies. Figure 2 provides the descriptions of each LASSI scale. LASSI includes statements asking users to describe their behavior or thoughts using a Likert scale. The assessment is a 60-question inventory that takes 10–15 min to complete. Each LASSI scale score was reported as a standardized percentile and scores were distributed into three groups: low (< 50th percentile), moderate (50th–75th percentile), and high (> 75th percentile) categories of perceived ability related to learning and study strategies [23]. Low to moderate perceived ability may indicate a need for development of more effective strategic learning [23].

Program GPA data (quarter 1 and quarter 2 GPA) and Quarter 1 course grades for Human Anatomy I and Physiology were available as part of program data records. Foundational anatomy and physiology courses can be challenging in DPT education; therefore, these individual course grades were included in the analysis.

### Statistical Methods

All data analyses were performed using SPSS v 27 with an alpha level of < 0.05. Demographic data was summarized with descriptive statistics. While the first measurement of the LASSI was taken during orientation (pre-LASSI), a second measurement of the LASSI was collected at the end of the 2nd quarter of the program for all students (post-LASSI). Mean scale percentile scores were used for comparisons of pre- and post-LASSI scores with a paired *t*-test. Cohen’s criteria were utilized to interpret effect-size for statistically significant findings: 0.2, small; 0.5, moderate; and 0.8, large effect [24].

Bivariate correlations (Spearman’s rho, $\rho$) described relationships between LASSI results and academic scores including human anatomy I, physiology, Quarter 1 GPA and Quarter 2 GPA. Interpretation of correlation strength was as follows: strong/high (0.70 to 1.0), moderate (0.50 to 0.70), low/weak (0.30 to 0.50), and negligible (0.0 to 0.30) [25].

## Results

### Participants

Data for pre-LASSI and post-LASSI scores as well as academic scores was collected for 259 matriculated DPT students. Table 1 summarizes demographic data. Participants were predominantly women (54%) and white (79%).

**Table 1:** Demographic summary, *n* = 259 DPT students

Demographic	Specifics	Total (percentage)
Sex	Female	140 (54%)
Race/ethnicity	Black	16 (6%)
	Pacific Islander	0 (0%)
	Hispanic	14 (5%)
	Asian	2 (< 1%)
	American Indian, Alaska native	1 (< 1%)
	White	205 (79%)
	Two or more	11 (4%)
First-generation college student		63 (24%)
Primary language other than English		6 (2%)
Physical therapist assistant		32 (12%)

### Pre- and post-LASSI differences

**Fig. 3:**
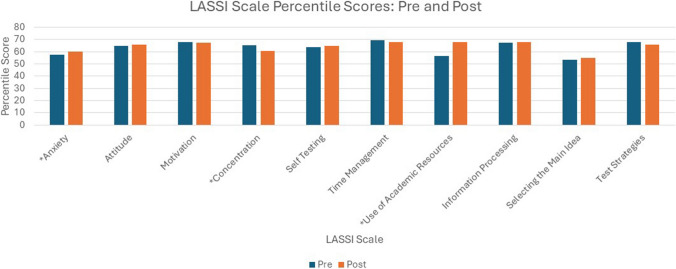
Percentile scores for each LASSI component at two points in time (pre, post). *Statistically significant differences in LASSI score, *p* < .05

Table 2 includes mean scores for each LASSI scale percentile ranking at matriculation (pre-LASSI) and the end of Quarter 2 (6 months later, post-LASSI). Mean percentile scores at both points in time indicate moderate perceived ability in each scale (range 53.3 to 69.6%). The standard deviations of all percentile rankings (both pre- and post-LASSI) were large, indicating wide variability in scores across individual students. Visual representation of mean scores at the two points in time is seen in (Fig. 3).

**Table 2:** Pre- and post-LASSI percentile scores

	Pre-LASSI: mean (sd)	Post-LASSI: mean (sd)	Difference (95% confidence interval)	*p* value^a^	Effect size^b^
*Anxiety*	57.41 (26.5)	61.07 (30.1)	3.66 (0.58, 6.74)	0.020*	0.145
*Attitude*	64.54 (22.7)	65.79 (23.7)	1.25 (− 1.61, 4.11)	0.390	
*Motivation*	67.89 (21.3)	67.15 (24.4)	− 0.741 (− 3.59, 2.10)	0.608	
*Concentration*	65.08 (22.5)	60.75 (25.7)	− 4.33 (− 6.97, − 1.69)	0.001*	0.201
*Self-Testing*	63.91 (24.2)	64.51 (24.4)	0.595 (− 2.15, 3.34)	0.670	
*Time Management*	69.59 (22.9)	68.05 (25.7)	− 1.54 (− 4.34, 1.25)	0.278	
*Use of Academic Resources*	56.47 (25.9)	53.26 (26.5)	− 3.21 (− 6.38, − 0.05)	0.047*	0.124
*Information Processing*	67.17 (23.6)	67.68 (24.7)	0.502 (− 2.30, 3.31)	0.725	
*Selecting the Main Idea*	53.31 (25.5)	54.93 (26.4)	1.62 (− 1.65, 4.89)	0.329	
*Test Strategies*	67.66 (22.2)	65.61 (25.8)	− 2.05 (− 5.26, 1.16)	0.211	

*DPT*, Doctor of Physical Therapy; *sd*, standard deviation

*Statistically significant, *p* < 0.05. ^a^Paired *t*-test analysis. ^b^Effect size estimates (Cohen’s *d*) are shared for statistically significant differences only

A paired *t*-test was used to examine change in LASSI scores for each of the ten scales. While there were outliers, none were greater than 1.5 box lengths from the edges of the box and no extreme outliers. All but two analyses did violate the assumption or normality (Shapiro–WIlk’s test); however, the paired *t*-test is robust and can handle these violations [26]. Small changes to percentile scores were noted across all LASSI scales, and five of ten scales showed lower post-LASSI percentile scores. Three of the ten scales had a statistically significant change and effect sizes were small (0.2 or less) [24]. *Anxiety* management scale improved percentile ranking by 3.66% (95% CI, 0.58, 6.74), *t*(258) = 2.340, *p* = 0.020. *Concentration* percentile rank lowered by 4.33% (95% CI, − 6.97, − 1.69), *t*(258) =  − 3.229, *p* = 0.001. *Use of Academic Resources* also decreased in percentile rank by 3.21% (95% CI, − 0.049, − 1.99), *t*(258) =  − 1.999, *p* = 0.047. Table 2 reports all paired *t*-test results and Fig. 3 shows mean scores of each scale at both points in time.

### Correlations

Correlations of pre- and post-LASSI with anatomy and physiology course grades and quarter GPAs are presented in Table 3. While there are several individual scores that have a statistically significant correlation, there are a few that have significant correlations across multiple academic scores. *Anxiety* management was significantly correlated with both course grades and both GPAs at both pre- and post-LASSI. Spearman rho ($\rho )$ values ranged from 0.246 to 0.407 for *Anxiety* management, and all correlations were stronger for the post-LASSI. *Test Strategies* was also significantly correlated with both course grades and GPAs for both pre- and post-LASSI. Spearman rho values ranged from 0.132 to 0.431 for this variable, and again, all correlations were stronger for the post-LASSI. *Motivation* and *Time Management* were statistically correlated to physiology and both Quarter 1 and Quarter 2 GPAs for the post-LASSI. *Information Processing* was also significantly correlated with anatomy and both Quarter 1 and Quarter 2 GPAs for the post-LASSI. *Selecting the Main Idea* was statistically correlated with anatomy, physiology, and both quarter GPAs for the post-LASSI. Table 3 reports for all correlation values of LASSI scores (pre and post) with anatomy and physiology course grades as well as DPT program GPA.

**Table 3:** Correlations of LASSI scores (pre and post) with DPT course grades and program GPA

	Quarter 1 Anatomy Course Grade^ϯ^	*p* value	Quarter 1 Physiology Grade ^ϯ^	*p* value	Quarter 1 GPA ^ϯ^	*p* value	Quarter 2 GPA ^ϯ^	*p* value
*Anxiety* management (pre)	0.321 (0.203, 0.429)	*p* < 0.001*	0.246 (0.124, 0.361)	*p* < 0.001*	0.265 (0.144, 0.378)	*p* < 0.000*	0.249 (0.127, 0.363)	*p* < 0.000*
*Anxiety* management (post)	0.391 (0.279, 0.492)	*p* < 0.001*	0.407 (0.296, 0.507)	*p* < 0.001*	0.359 (0.244, 0.463)	*p* < 0.000*	0.390 (0.278, 0.491)	*p* < 0.000*
*Attitude* (pre)	− 0.038 (− 0.163, 0.088)	*p* = 0.540	− 0.070 (− 0.194, 0.056)	*p* = 0.262	0.021 (− 0.146, 0.104)	*p* = 0.731	0.026 (− 0.100, 0.151)	*p* = 0.675
*Attitude* (post)	0.046 (− 0.080, 0.170)	*p* = 0.465	0.122 (− 0.003, 0.244)	*p* = 0.051	0.095 (− 0.030, 0.218)	*p* = 0.125	0.128 (0.003, 0.250)	*p* = 0.039*
*Motivation* (pre)	0.026 (− 0.100, 0.151)	*p* = 0.674	0.010 (− 0.116, 0.135)	*p* = 0.874	0.033 (− 0.093, 0.158)	*p* = 0.598	0.037 (− 0.089, 0.162)	*p* = 0.555
*Motivation* (post)	0.110 (− 0.016,0.232)	*p* = 0.078	0.140 (0.015, 0.261)	*p* = 0.024*	0.148 (0.023, 0.268)	*p* = 0.017*	0.148 (0.023, 0.269)	*p* = 0.017*
*Concentration* (pre)	0.056 (− 0.071, 0.180)	*p* = 0.373	0.002 (− 0.124, 0.127)	*p* = 0.980	0.072 (− 0.054, 0.196)	*p* = 0.246	0.017 (− 0.109, 0.142)	*p* = 0.787
*Concentration* (post)	0.072 (− 0.054,0.196)	*p* = 0.250	0.101 (− 0.025, 0.223)	*p* = 0.107	0.128 (0.002, 0.249)	*p* = 0.040*	0.121 (− 0.005, 0.243)	p = *0.052*
*Self-Testing* (pre)	0.066 (− 0.060, 0.191)	*p* = 0.287	0.014 (− 0.112, 0.139)	*p* = 0.823	0.042 (− 0.084, 0.166)	*p* = 0.502	0.039 (− 0.087, 0.164)	*p* = 0.532
*Self-Testing* (post)	0.045 (− 0.081, 0.170)	*p* = 0.472	0.072 (− 0.054, 0.196)	*p* = 0.251	0.059 (− 0.067, 0.184)	*p* = 0.341	0.124 (0.002, 0.246)	*p* = 0.046*
*Time Management* (pre)	0.020 (− 0.106, 0.145)	*p* = 0.752	0.047 (− 0.080, 0.171)	*p* = 0.456	0.113 (− 0.012, 0.235)	*p* = 0.069	0.098 (− 0.028, 0.221)	*p* = 0.115
*Time Management* (post)	0.051 (− 0.075, 0.176)	*p* = 0.412	0.131 (0.005, 0.252)	*p* = 0.036*	0.141 (0.016, 0.262)	*p* = 0.023*	0.170 (0.045, 0.289)	*p* = 0.006*
*Use of Academic Resources* (pre)	− 0.105 (− 0.228, 0.021)	*p* = 0.093	− 0.151 (− 0.272, − 0.026)	*p* = 0.015*	− 0.131 (− 0.252, − 0.005)	*p* = 0.036*	− 0.095 (− 0.218, 0.31)	*p* = 0.127
*Use of Academic Resources* (post)	− 0.080 (− 0.204, 0.046)	*p* = 0.198	− 0.029 (− 0.154, 0.098)	*p* = 0.649	− 0.073 (− 0.197, 0.052)	*p* = 0.239	− 0.002 (− 0.127, 0.124)	*p* = 0.979
*Information Processing* (pre)	0.032 (− 0.094, 0.157)	*p* = 0.606	0.057 (− 0.070, 0.181)	*p* = 0.365	0.051 (− 0.075, 0.175)	*p* = 0.413	0.067 (− 0.058, 0.191)	*p* = 0.279
*Information Processing* (post)	0.154 (0.028, 0.274)	*p* = 0.014*	0.167 (0.042, 0.286)	*p* = 0.007*	0.173 (0.048, 0.292)	*p* = 0.005*	0.112 (− 0.013, 0.235)	*p* = 0.071
*Selecting the Main Idea* (pre)	0.143 (0.018, 0.264)	*p* = 0.021*	0.037 (− 0.089, 0.162)	*p* = 0.553	0.094 (− 0.032, 0.216)	*p* = 0.133	0.080 (− 0.046, 0.204)	*p* = 0.199
*Selecting the Main Idea* (post)	0.250 (0.129, 0.364)	*p* < 0.001*	0.272 (0.151, 0.384)	p < 0.001*	0.256 (0.135, 0.369)	*p* < 0.005*	0.284 (0.165, 0.395)	*p* < 0.005*
*Test Strategies* (pre)	0.171 (0.046, 0.290)	*p* = 0.006*	0.132 (0.006, 0.253)	*p* = 0.035*	0.197 (0.073, 0.315)	*p* = 0.001*	0.141 (0.015, 0.261)	*p* = 0.024*
*Test Strategies* (post)	0.357 (0.242, 0.462)	*p* < 0.001*	0.388 (0.276, 0.490)	*p* < 0.001*	0.370 (0.256, 0.473)	*p* < 0.005*	0.431 (0.323, 0.528)	*p* < 0.005*

*LASSI*, Learning and Study Strategies Inventory; *DPT*, Doctor of Physical Therapy; *GPA*, grade point average

*Statistically significant, *p* < 0.05; ^ϯ^Spearman’s rho ($\rho$) and 95% confidence interval

## Discussion

The LASSI is a self-report questionnaire used to inform users on self-regulated learning strategies. LASSI can provide learners (and educators) with an awareness of strengths and weakness related to strategic learning. LASSI results can be used as a pre-post measure of learning strategy intervention or to drive educational intervention [23]. LASSI scores showed small percentile ranking change at the post-LASSI after the science of learning course and 2 quarters of the DPT program. For the baseline pre-LASSI scores at matriculation, all mean percentile scores were in the moderate range (between 50 and 75th percentile), indicating learners should consider strategic learning skills improvement to maximize success [23]. In the DPT program described in the current study, a science of learning course provided students with information on evidence-based learning strategies including those associated with strategic learning principles (dual coding, spaced practice, self-awareness, focus, environment for learning, emotion associated with learning, sleep, and stress management) and a master adaptive learning model (promotion of critical thinking and self-reflection). However, there is no training geared directly toward a specific LASSI scale. After the science of learning course and two quarters of the 2-year hybrid DPT program, mean percentile rankings remained in the moderate range. Findings showed three of ten scales had a significant change over time, but effect sizes were small, and two of these showed a lower percentile ranking on the post-LASSI. If mean LASSI scores were in the moderate range at both points in time, this indicates learners should still consider bolstering strategic learning for future coursework. Educators should consider more personalized strategic learning interventions such as coaching/mentoring, establishing learning plans, and/or goal setting around the individual areas of need. An examination of LASSI scores and discussion of the items that are below the 50th percentile could be a first step in this individualized coaching approach.

There is limited literature reporting on the use of LASSI over time, either with or without an intervention. The current study utilized a different sample of DPT students from a 2-year hybrid DPT program which may yield different results to other samples. Scales and Vallabhjosula [17] investigated change in LASSI scores over time in 211 entry-level DPT students. The pre-LASSI was at matriculation, there was no intervention, and the post-LASSI was 1 year into the DPT program. The authors of this study found similar results to findings in the current study; all LASSI scales scored below the 75th percentile in the pre- and post-LASSI assessment periods. Significant increases were noted in four of the ten scales (including *Anxiety* management, which also increased in the current study), and two scales showed significant decline (*Attitude* and *Concentration;* in the current study, *Concentration* and *Use of Academic Resources* decreased). The findings from Scales and Vallabhiousla et al. are similar to results of the current study where an intentional science of learning course was provided.

Pucillo and Perez [27] investigated the change in LASSI and the relationship to GPA at the end of the first year for DPT or occupational therapy students after an educational intervention course specifically related to student LASSI scale scores. The authors report significant changes in pre- to post-LASSI in the scales of *Concentration*, *Self-testing*, *Test Strategies*, and *Time Management*. Their finding of a positive significant change in *Concentration* with a small effect size (*d* = 0.17) is in direct contrast to findings in the current study where there was a significant negative effect (*d* = 0.20). Sisa et al. [28] also examined change in LASSI scores in 3rd year medical students (*n* = 107) after five workshops designed around coaching the study strategies from LASSI. These authors found all scale scores increased except *Motivation* [28]. An interesting difference between current study results and those found by Sisa et al. is the initial scores (the pre-test scores). In the medical student baseline, LASSI scores were below the 50th percentile for all but two scales (*Information Processing*, *Self-Testing*) [28], and in the current study of DPT students, all baseline scores were above the 50th percentile. This difference implies there was more room for scales to improve over time in the study by Sisa et al. [28].

It is not clear why some LASSI scale scores declined over time in the current study or in other research. However, researchers can speculate. There is a chance students learned something new about the strategic learning principles during the science of learning course, and this changed their self-reflection or perception of their ability in various areas. It is also possible that the strategies they perceived as strengths in undergraduate education shifted once they had graduate level DPT education, especially at the pace of a 2-year DPT program.

The second aim of the current study was to examine relationships between LASSI scale scores and academic grades (anatomy and physiology course grades and program GPA). Previous research has looked at the relationship of LASSI scores to DPT admissions data, reporting no strong correlations with this data [29]. Knowing that admissions data also show weak to negligible correlations to DPT program GPA [30], it is possible program GPAs do have a relationship to LASSI scales. Correlations of LASSI scores to DPT program GPA in the current study were low to negligible, yet all correlations, except for *Use of Academic Resources*, were stronger in the post-LASSI, after the science of learning course and two quarters of the DPT program. These results are similar to observation in medical students, where the post-LASSI assessments given at the beginning of year 2 had more frequent significant correlations than the pre-LASSI given at the beginning of medical school [17]. Another study examining LASSI scores as related to academic performance (categories of low and high performers in medical school) found *Anxiety*, *Motivation* and *Test Strategies* were different in students who had an A average versus those with a C average in their biomedical science courses [31].

*Anxiety* management and *Test Strategies* were the only two LASSI scales with a significant correlation to GPA for both the pre- and post-LASSI in the current study. *Anxiety* management was also the scale that statistically improved over time, and other literature supports that finding [20]. A student’s ability to manage anxiety can have an impact on academic performance. Educators, specifically for healthcare relate fields, should continue to consider anxiety management interventions for student support, including the benefit to learning [32]. A 2021 meta-analysis examined LASSI scores and academic outcomes in higher education students reporting the *Motivation* scale which had the strongest correlation to positive academic outcomes [33]. Results of the current study also showed that the *Motivation* scale was positively and significantly correlated to physiology and both quarter GPAs in the post-LASSI assessment. Motivation is a pillar of self-regulated learning and is impactful in academic success [34, 35].

Academic faculty should consider discussing motivation with students alongside intentional efforts to foster and support motivation to reach program and personal learner goals.

## Limitations and Future Research

Data was collected from one 2-year hybrid DPT program at a single institution which may not be representative of all hybrid DPT programs and may be different from residential DPT programs. There was no control group that did not receive the science of learning course for comparison. The administration of LASSI does have a financial cost per student that may impact widespread utilization. LASSI is a self-report tool examining elements of strategic learning that are likely contextually dependent. It is possible students would score themselves differently under different circumstances or at different points in the DPT curriculum. The stability and responsiveness of the LASSI over time have not been studied or reported in the literature, and therefore, it is unclear if this tool is responsive to change or what level of change indicates meaningful change.

Future research should examine intervention directed at promoting strategic learning principles as well as psychometric properties of the LASSI. Given the number of courses students take during the first 2 quarters of the program, it is not possible to relate changes in LASSI scores specifically to the science of learning course. Further examination of student perception of change in the use of strategic learning principles may help explain the decline or change of some scale scores. The small changes in LASSI over time and weak to moderate correlation strengths may suggest a time component that was not measured. It is possible a follow-up assessment of LASSI later in the curriculum would yield different results. Future research could consider different points in time for a follow-up assessment of LASSI.

## Conclusion

LASSI mean percentile scores were in the moderate aptitude range for all scales at baseline (matriculation into the DPT program) and 6 months later after a science of learning course and two quarters in the DPT program. This moderate range score indicates an overall need for support and growth in strategic learning. While LASSI scores did change over time, the change was small with only three scales out of ten having a significant difference and two of those scale percentiles were lower in the post-LASSI assessment. All effect sizes were small. LASSI correlations with DPT anatomy and physiology course grades and GPA for Quarter 1 and Quarter 2 were positive; however, overall strength was weak to negligible. Future studies should include psychometric analysis of the LASSI in DPT students. Further examination of strategic learning and other self-regulated learning components is warranted as well as examination of student support for growth and the promotion of academic achievement.

## Supplementary Information

Below is the link to the electronic supplementary material.

Supplementary file1 (XLSX 36 KB)

## Data Availability

Available upon request from corresponding author.

## References

[1] Weinstein CE, Acee TW, Jung J. Self-regulation and learning strategies. New Dir Teach Learn. 2011;2011:45–53. 10.1002/tl.443.

[2] Banarjee P, Kumar K. A study on self-regulated learning and academic achievement among the science graduate students. Int J Multidiscip Approach Stud. 2014;1:329–42 ISSN: 2348 – 537X.

[3] Broadbent J, Sharman S, Panadero E, Fuller-Tyszkiewicz M. How does self-regulated learning influence formative assessment and summative grade? Comparing online and blended learners. Internet High Educ. 2021;50:100805. 10.1016/j.iheduc.2021.100805.

[4] Ergen B, Kanadli S. The effect of self-regulated learning strategies on academic achievement: a meta-analysis study. Eurasian J Educ Res. 2017;17:55–74. 10.14689/ejer.2017.69.4.

[5] Yan Z. Self-assessment in the process of self-regulated learning and its relationship with academic achievement. Assess Eval High Educ. 2020;45:224–38. 10.1080/02602938.2019.1629390.

[6] Panadero E. A review of self-regulated learning: six models and four directions for research. Front Psych. 2017;8:1–28. 10.3389/fpsyg.2017.00422. 10.3389/fpsyg.2017.00422 PMC5408091 28503157

[7] Lajoie SP, Gube M. Adaptive expertise in medical education: accelerating learning trajectories by fostering self-regulated learning. Med Teach. 2018;40:809–12. 10.1080/0142159X.2018.1485886. 30033791 10.1080/0142159X.2018.1485886

[8] Cupido N, Ross S, Lawrence K, Bethune C, Fowler N, Hess B, et al. Making sense of adaptive expertise for frontline clinical educators: a scoping review of definitions and strategies. Adv Health Sci Educ Theory Pract. 2022;27:1213–43. 10.1007/s10459-022-10176-w. 36302908 10.1007/s10459-022-10176-w

[9] Inzlicht M, Werner KM, Briskin JL, Roberts BW. Integrating models of self-regulation. Annu Rev Psychol. 2021;72:319–45. 10.1146/annurev-psych-061020-105721. 33017559 10.1146/annurev-psych-061020-105721

[10] Geller J, Toftness AR, Armstrong PI, Carpenter SK, Manz CL, Coffman CR, et al. Study strategies and beliefs about learning as a function of academic achievement and achievement goals. Memory. 2018;26:683–90. 10.1080/09658211.2017.1397175. 29096586 10.1080/09658211.2017.1397175

[11] Blasiman RN, Dunlosky J, Rawson KA. The what, how much, and when of study strategies: comparing intended versus actual study behaviour. Memory. 2017;25:784–92. 10.1080/09658211.2016.1221974. 27561889 10.1080/09658211.2016.1221974

[12] Rea SD, Wang L, Muenks K, Yan VX. Students can (mostly) recognize effective learning, so why do they not do it? J Intell. 2022;10:127. 10.3390/jintelligence10040127. 10.3390/jintelligence10040127 PMC9781761 36547514

[13] Madan CR. Using evidence-based learning strategies to improve medical education. Med Sci Educ. 2023;33:773–6. 10.1007/s40670-023-01798-9. 37501813 10.1007/s40670-023-01798-9 PMC10368606

[14] Wolters CA, Won S. Validity and the use of self-report questionnaires to assess self-regulated learning. In: Schunk DH, Greene JA, editors. Handbook of self-regulation of learning and performance. 2nd ed. New York: Routledge; 2018. p. 307–22.

[15] Khalil MK, Williams SE, Gregory Hawkins H. Learning and study strategies correlate with medical students’ performance in anatomical sciences. Anat Sci Educ. 2017;11:236–42. 10.1002/ase.1742. 28940743 10.1002/ase.1742

[16] Pucillo EM, Perez G. Metacognition and self-regulation influence academic performance in occupational and physical therapy students. J Occup Ther Educ. 2023;7:4. 10.26681/jote.2023.070104.

[17] Khalil MK, Hawkins HG, Crespo LM, Buggy J. The relationship between learning and study strategies inventory (LASSI) and academic performance in medical schools. Med Sci Educ. 2017;27:315–20. 10.1007/s40670-017-0400-x.

[18] Stephens K. The LASSI as a measure of doctoral of physical therapy students study skills and its relationship to PEAT and NPTE. University of Missouri--Columbia; 2022. Thesis; 10.32469/10355/91691

[19] Zhou Y, Graham L, West C. The relationship between study strategies and academic performance. International J Med Educ. 2016;7:324–32. 10.5116/ijme.57dc.fe0f. 10.5116/ijme.57dc.fe0f PMC5056023 27718497

[20] Scales MH, Vallabhajosula S. Learning and study strategies of students in the first year of an entry-level physical therapist program. J Phys Ther Educ. 2023;37:132–7. 10.1097/JTE.0000000000000275. 38478827 10.1097/JTE.0000000000000275

[21] Cutrer W, Pusic M, Gruppen LD, Hammoud MM, Santen SA. The master adaptive learner. Elsevier Health Sciences; 2019.

[22] Weinstein, Claire Ellen, David R Palmer, Taylor W Acee. Users Manual, LLO, Learning and Study Strategies Inventory for Learning Online. 2020. Available from: https://wwwhhpublishing.com/ap/_assessments/LLO_Users_Manual.pdf. Accessed 09 Dec 2022.

[23] Weinstein CE, Palmer DR, Acee TW. LASSI User’s Manual, Learning and Study Strategies Inventory 3rd Edition. H&H Publishing Company, Inc, Clearwater, FL; 2016. Available from: https://www.hhpublishing.com/LASSImanual.pdf. Accessed 9 Dec 2022.

[24] Cohen J. The concepts of power analysis. In: Cohen J, editor. Statistical power analysis for the behavioral sciences. New York: Routledge; 1988. p. 1–17; 10.4324/9780203771587.

[25] Mukaka MM. Statistics corner: a guide to appropriate use of correlation coefficient in medical research. Malawi Med J. 2012;24:69–71. 23638278 PMC3576830

[26] Statistics L. Multiple regression using SPSS Statistics. Statistical tutorials and software guides. 2015. Available from: https://statistics.laerd.com/. Accessed 2 Sept 2024.

[27] Pucillo EM, Perez G. Training and instruction of learning and study strategies improve academic performance in rehabilitation students. Internet J Allied Health Sci Pract. 2023;21:13.

[28] Sisa I, Garcés MS, Crespo-Andrade C, Tobar C. Improving learning and study strategies in undergraduate medical students: a pre-post study. Healthcare (Basel). 2023. Jan 28;11(3)375. 10.3390/healthcare11030375. 10.3390/healthcare11030375 PMC9914150 36766950

[29] Minahan CA, Reynolds B, Martin JG, Seale J. Strategic learning strategies of doctor of physical therapy students. Journal of Physical Therapy Education. Published online ahead of print Oct 25,2024. Available from: http://ovidsp.ovid.com/ovidweb.cgi?T=JS&PAGE=reference&D=ovftz4&NEWS=N&AN=00001416-900000000-99724 10.1097/JTE.0000000000000345 39298546

[30] Reynolds B, Unverzagt C, Koszalinski A, Gatlin R, Seale J, Gagnon K, et al. Predictors of success on the national physical therapy examination in 2 US accelerated-hybrid doctor of physical therapy programs. J Phys Ther Educ. 2022;36:225. 10.1097/JTE.0000000000000227.

[31] Khalil MK, Williams SE, Hawkins HG. The use of learning and study strategies inventory (LASSI) to investigate differences between low vs high academically performing medical students. Med Sci Educ. 2019;30:287–92. 10.1007/s40670-019-00897-w. 34457669 10.1007/s40670-019-00897-w PMC8368407

[32] Parsons D, Gardner P, Parry S, Smart S. Mindfulness-based approaches for managing stress, anxiety and depression for health students in tertiary education: a scoping review. Mindfulness. 2022;13:1–16. 10.1007/s12671-021-01740-3. 34539929 10.1007/s12671-021-01740-3 PMC8435111

[33] Fong CJ, Krou MR, Johnston-Ashton K, Hoff MA, Lin S, Gonzales C. LASSI’s great adventure: a meta-analysis of the learning and study strategies inventory and academic outcomes. Educ Res Rev. 2021;34:100407. 10.1016/j.edurev.2021.100407.

[34] Schunk DH, Zimmerman BJ. Motivation and self-regulated learning: theory, research, and applications. New York: Taylor & Francis Group, LLC; 2012.

[35] Zimmerman BJ. Investigating self-regulation and motivation: historical background, methodological developments, and future prospects. Am Educ Res J. 2008;45:166–83. 10.3102/0002831207312909.

